# Enhancing Job Performance: The Critical Roles of Well-Being, Satisfaction, and Trust in Supervisor

**DOI:** 10.3390/bs14080688

**Published:** 2024-08-08

**Authors:** Yiting Yang, Bojan Obrenovic, Daniel W. Kamotho, Danijela Godinic, Dragana Ostic

**Affiliations:** 1 School of Business and Management, The Hong Kong University of Science and Technology, Hong Kong 999077, China; yyangfs@outlook.com; 2 The Department of Management, Entrepreneurship and Digital Transformation, Zagreb School of Economics and Management, 10000 Zagreb, Croatia; 3 School of Business and Management, Q University, Almaty 050026, Kazakhstan; 4 Department of Accounting, Economics and Finance, Namibia University of Science and Technology, Windhoek 9000, Namibia; dwkamotho@yahoo.com; 5 Faculty of Humanities and Social Sciences, University of Zagreb, 10000 Zagreb, Croatia; danijela.godinic5@gmail.com; 6 School of Finance, Jiangsu University, Zhenjiang 212013, China; dostic@ujs.edu.cn

**Keywords:** job performance, employee psychological well-being, job satisfaction, trust in supervisor, in-role performance

## Abstract

This empirical study investigated the associations between psychological well-being, job satisfaction, trust in supervisor, and job performance. Data were collected from 277 company employees in Bahrain through online questionnaires and analyzed using structural equation modeling (SEM). The purpose of the study is to examine the relationships between psychological well-being, job satisfaction, trust in supervisor, and job performance through the use of a theoretical framework that synthesizes prominent models in the field of organizational psychology. The research findings indicate a positive influence of psychological well-being on both job satisfaction and job performance. Notably, job satisfaction plays a mediating role in the relationship between psychological well-being and job performance. The study contributes to the existing body of knowledge by offering an integrated approach to examining the intricate connections between psychological well-being, job satisfaction, trust in supervisor, and job performance, which all are crucial for creating a sustainable workplace environment and outcomes. Practical implications highlight the need for organizations to prioritize employee psychological well-being through initiatives such as wellness programs and supportive work environments, as these initiatives directly influence job satisfaction and performance. Job satisfaction acts as a significant mediator, emphasizing the importance of fair compensation, recognition, and professional development in enhancing job satisfaction.

## 1. Introduction

In the last decade, the managerial and organizational literature on job performance has advanced with the creation of novel theoretical approaches. The latest progress includes organizational and psychosocial models concerning the post-COVID-19 paradigmatic transition, both to remote work policy and organizational operational system changes [1,2]. Notwithstanding the technological and AI implementations, human capital remains the central resource that most organizational activities rely upon [3]. However, companies may lose their competitive edge if management fails to motivate their employees and coordinate their operations to benefit from technological resources [4]. For this reason, understanding human motivation [5], together with its intrinsic and extrinsic dimensions, remains a fundamental task for management and behavioral studies [6].

As job satisfaction has been recognized as an antecedent of job performance [7,8,9,10], organizations are increasingly employing diverse incentives to improve their employees’ occupational and career satisfaction. They apply different techniques, such as rewards, bonuses, recognition, and promotion, to make the employees feel secure, appreciated, and noticed [11,12].

High-level-trust organizations benefit from willing and compliant employees, which leads to better financial performance and increased profitability [13]. In a high-trust work setting, employees report 74% less stress, 106% more engagement, and 50% greater productivity [14]. Moreover, the profound connection between psychological well-being and work performance is evident in the fact that in a reliable and trustful environment, employees report greater life satisfaction and less burnout [15]. However, when employees experience high-level uncertainty, they are easily triggered by work stressors and are thus more likely to experience decreased productivity and high turnover. Thus, trust is a paramount factor in the perceptions of well-functioning organizations [16].

If the underlying psychological mechanisms are understood correctly and human resources are managed accordingly, employees become a source of competitive advantage [17,18]. Under proper governance and given enough autonomy, employees can create profitable opportunities for success through innovation [19,20].

The current study is valuable given that the understanding of the processes that lead from psychological satisfaction to job performance is scarce [21]. Therefore, this paper presents a direct response to the need for further investigation to support the relationships and possible mediators that would improve organizational work efficiency [22,23].

This current study closely follows the paradigm of United Nations Sustainable Development Goal (SDG) 3, specifically the objective of proposing new initiatives and agendas for ensuring healthy lives and promoting the wellbeing at all ages [24]; and SDG 8 Decent work and economic growth, including the promotion of development-oriented policies that support productive activities. Some of the vital targets in this context encompass enhancing employee well-being and implementing mindfulness practices. Based on the empirical evidence, we characterize different aspects of sustainability and psychological health drivers, upon which wellness and support programs can be formed. We contend that content employees are more productive, i.e., workers who feel supported, valued, and encouraged by the organization are more likely to experience satisfaction, which will reduce turnover intention, improve their performance, and bring revenue to the organization [25]. With this in mind, current research advocates for implementing a series of measures that will supply individuals with sufficient resources to increase resilience and facilitate a safe and rewarding work environment [26]. The SDG objectives are of central interest in much of the current research in the fields of occupational health, organizational psychology, and behavioral sciences [27]. In order to address the SDG 3 aims outlined above, our study reports on determinants of job satisfaction and job performance, both of which are indicative of well-balanced and self-actualized individuals, and ultimately aligns with global efforts to create healthier societies [28].

To bridge this research gap, the present paper develops a unique framework based on the well-grounded Maslow’s hierarchy of needs model and Conservation of Resources Theory. We integrate the main tenets and combine them to extend the explanation of employees’ psychological well-being in terms of job-related satisfaction motives to establish a connection with job performance. Our primary aim is to deepen the comprehension of the underpinnings generating positive organizational outcomes. The study lays the groundwork for improving the study of job satisfaction and job performance in a Middle East business context. Furthermore, this paper explains how job satisfaction stemming from a positive assessment of the work situation effectively creates a productive workforce, thus enhancing organizational productivity and generating revenue, decreasing turnover rates and reducing distress.

## 2. Literature Review

### 2.1. Key Concept Overview

#### 2.1.1. Psychological Well-Being

Psychological health is a global challenge and a focus of scholarly attention, particularly in relation to socioeconomic adversities and preventive practices. In an organizational context, psychological well-being is a multifaceted construct closely related to employees’ psychological plasticity, adaptability, motivation, social exchange, work experience, satisfaction, and productivity [29,30,31]. Mohan and Lone (2021) have framed well-being through four categories, namely, physical self-care, personal purpose, mental reactiveness to pressures, and emotional stability [32]. Furthermore, from an organizational perspective, psychological well-being refers to employee mental health, self-regulation ability, and resilience under the varying conditions of workplace stability or uncertainty [32]. Therefore, it is influenced by psychological flexibility and organizational stability [33]. Psychological well-being is shaped by psychosocial and structural factors such as cognitive ability and organizational support. Generally, it is enhanced through mindfulness and stress management frameworks, inclusion, safety, and cooperation [34,35]. In the current study, it is contingent on the level of autonomy, satisfaction, professional development, and supervisor support [36]. It is based on interaction between individual, interpersonal, and structural characteristics within the work setting.

#### 2.1.2. Job Satisfaction

Job satisfaction is considered to be significantly context-dependent [37]. The managerial literature stresses that stimulating and attaining job satisfaction is an essential organizational priority because it helps to decrease the turnover rate and improve organizational performance. Employers prefer to accommodate their workers because workplace happiness is related to their job market behavior, such as efficiency, resignation, and absenteeism [38]. Research shows that emotional well-being derived from enjoyment at work has a significant positive effect on job satisfaction [39]. Recent methods focus on overcoming the problems related to SDG 3 by proposing different schemes for improving the organizational culture favoring individuals’ well-being and happiness [26]. We examine previous work and propose a new conceptual framework committed to advancing employee psychological well-being, which in turn fosters a productive, healthy, and sustainable workforce [28,40].

#### 2.1.3. Job Performance

In addition to being the ultimate organizational objective, performance concerns both the effort enacted and the results obtained. Thus, employee performance can be referred to as the employee’s expected work-associated deeds that also encompass how activities are completed. Job performance was previously defined in terms of employees’ aggregated financial and nonmonetary added value brought to the organization [41]. Worker performance involves measurable behaviors that appear during the work process and reflect the company’s objectives [42]. Job performance can lead to numerous extrinsic rewards, such as bonuses, promotions, and recognition [43]. Notwithstanding the digital, strategic, and capital resources contributing to organizational sustainability, human shareholders remain the driving force of organizational performance. Performance was previously defined in terms of task completion success levels over a determined period compared to standard results or level of completion of target objectives [8]. Furthermore, job performance is characterized as an output result stemming from engagement in business undertakings [44]. Employee performance arises when employees carry out duties based on education, knowledge, effort, and opportunity [45]. Overall job performance is predicated on the amount of effort individual employees invest in performing tasks and obligations [46,47]. Coupled with organizational strategy, satisfaction, and economy, work increases performance [48].

#### 2.1.4. Trust in Supervisor

Since workers are highly dependent upon superiors in their work life, supervisors play a crucial role in forming employees’ views of their working conditions [49,50,51]. Supervisor support has a fundamental influence on well-being [52,53]. The supervisor’s availability, work support, and consultation buffer them from the adverse effects of work stressors [54].

Trust in the supervisor can be described as a psychological condition encompassing the willingness to acknowledge vulnerability based on the belief in the supervisor’s competence and abilities to control organizational outcomes [55,56]. Trust in the supervisor is fundamental in influencing individuals’ social capital through a feedback loop [57,58]. A rigorously discussed theme with a substantial consensus regarding its importance to the organization has been the interpersonal trust between supervisors and subordinates [59,60]. Trust provides the foundation for a positive working atmosphere between superiors and subordinates, promotes subordinates’ improved performance, and enhances growth opportunities within an organization. At times, it is even more meaningful than the competency [61]. Trust in supervisor contributes significantly to citizenship behavior and psychological well-being. It can be argued that employees receiving adequate organizational support will share a common mission and vision to support organizational objectives, which implicitly includes an increase in performance [62]. The correlation between trust in supervisor, psychological well-being, and job satisfaction is of interest due to the significant alignment with SDG 3 goals, particularly concerning the provision of encouraging and empowering a work environment [26] based on supportive supervisory relationships, which ultimately results in individual, and consequently organizational, well-being [27].

### 2.2. Theoretical Background and Research Model Development

**Figure 1:**
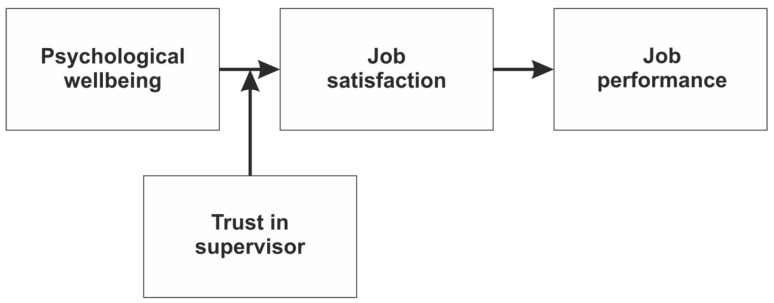
Research model.

Building on Maslow’s Theory of Needs and Conservation of Resources Theory, we have developed a unique theoretical framework for understanding the complex interplay between psychosocial antecedents to the supportive sustainable workplace environment. Furthermore, our novel research model explains how fulfilling fundamental needs and conserving critical resources results in favorable outcomes, such as improved psychological well-being, increased job satisfaction, and enhanced performance (Figure 1).

#### 2.2.1. Maslow’s Theory of Needs

Maslow’s Theory of Needs (1943) is a prominent framework for explaining human motivation which, in the context of the current study, provides a useful model for capturing complex relationships between psychological well-being, job satisfaction, trust in supervisor, and job performance [63]. In line with the theory, psychological well-being is contingent upon fulfilling the need for safety in a supportive work setting, while esteem needs are fulfilled by way of recognition. By gratifying lower level needs, job satisfaction is likely to be achieved. Furthermore, upon reassuring basic needs, higher-order needs for esteem and self-actualization can be pursued. When trustworthy and reliable, the supervisor is intimately related to a safe work environment and fulfilling the need for belonging. Trust in the supervisor boosts psychological well-being and, through it, job satisfaction and performance. Accordingly, satisfied and confident individuals will strive to perform better.

#### 2.2.2. Conservation of Resources Theory

According to Hobfoll’s Conservation of Resources (COR) Theory (1989), individuals strive to obtain, retain, and protect resources, whether tangible or intangible [64]. Psychological well-being, job satisfaction, and trust in supervisors all supply individuals with valuable resources and are deemed desirable. Psychological well-being is fundamental for individuals carrying out daily activities. Mindfulness and wellness programs procured by organizations as supportive entities help employees accumulate and conserve psychological resources, thus increasing job satisfaction. Job satisfaction mediates the relationship between psychological well-being and performance. As specified by COR, happy employees are more resilient and thus able to invest additional energy into procuring further supply, which leads to sustained job performance. Trust is essential as it ensures stability, resilience, and facilitates channeling key resources into productive activities while buffering from stressors such as work–life imbalance or uncertainty. A reliable supervisor encourages such resilience by supporting and rewarding, which results in increased well-being and satisfaction. Organizations with comprehensive resource-building strategies will more likely generate improved sustainability.

Moderate pressure at work is beneficial for psychological health, as it stimulates workers to grow and build resilience by undertaking challenges [65], whereas excessive work pressure negatively impacts employees’ well-being and, consequently, their job satisfaction and work outcomes [66]. Job functions as an essential source of tangible and intangible resources given that it procures both monetary and non-monetary benefits. For instance, work training significantly increases individuals’ skills, knowledge, and cognitive ability [67,68]. Psychological well-being is related to purpose, self-acceptance, sense of achievement, and mastery [69], while a lack of self-esteem and depression are related to low productivity and dissatisfaction in the workforce and mental well-being [70,71]. The relationship between work and mental health was especially made evident in the face of fear of socioeconomic repercussions during the pandemic and in the post-pandemic context [72,73]. Job loss, downsizing, and physical and social restrictions proved to have a severely adverse effect on psychological well-being [74,75,76]. Psychologically balanced employees generally find work meaningful, form better leader–member exchanges, and are more motivated to further their careers [77]. Robust organizational support elevates psychological empowerment, and it was previously found that psychological empowerment has a favorable effect on psychological well-being and ultimately increases job satisfaction [78,79]. Psychological well-being is closely related to work–life balance, meaning that positive employees have a better management of professional and personal domains, experience less conflict and adversity, and are more resilient to dealing with work–family conflict [80,81]. Furthermore, happy employees experience less work stress and job burnout [82]. A stream of studies found that pronounced job enjoyment influences job satisfaction. When comparing our results to those of older studies, it must be pointed out that the correlation between psychological well-being and job satisfaction is well established in the literature [83]. Studies suggest that happy employees tend to perform better than dissatisfied employees do, and they are usually more successful and socially engaged [3,84]. The proposed hypothesized relationship between psychological well-being and job satisfaction underlines just how important providing empowerment and stress management practices are to reaching the SDG objectives, namely SDG 3—ensuring healthy lives and promoting the wellbeing at all ages, and SDG 8—Decent work and economic growth, respectively [24,85]. One of the main characteristics of psychological well-being is its inherently phenomenological nature; one perceives that one feels satisfied only when one subjectively believes in his satisfaction [86]. In other words, perceiving a job as satisfactory is prompted by positive emotions, thus fueling satisfaction. Consequently, workers’ psychological well-being is likely to have a positive impact on personal and occupational development and growth.

Therefore, the following hypothesis is suggested:

**H1:**  *Psychological well-being has a significant positive impact on job satisfaction.*

Productivity resulting from employees’ effectiveness has always been paramount to organizations. When trying to ensure the best performance outcomes, it is essential to consider workers’ job satisfaction [87,88]. Since intentionally dissatisfied employees may hinder organizational efforts and objectives to express and emphasize their disappointment and disapproval of the organizational climate and leadership, satisfaction becomes central to improving organizations’ competitive advantage [89]. The relationship between job satisfaction and job performance is well documented [90]. Satisfied employees score higher on job-related tasks, and their attitude toward work is much more favorable than unsatisfied employees [8,91]. Psychologically well and satisfied employees take action to achieve their tasks in accordance with regular restrictions of accessible resources [92]. Thus, the following hypothesis is suggested:

**H2:**  *Job satisfaction has a positive impact on job performance.*

Trust enables supervisors to generate positive outcomes among subordinates, enhancing the quality of communication between the overseer and the subordinate. For example, when workers invest their time, energy, and resources in completing a task or project, supervisors are required to appreciate and reward their efforts accordingly [93,94]. Therefore, if workers assume that their superiors compensate them adequately, they will respond with greater vigor, dedication, and engagement [95,96]. Supervisors should have the necessary knowledge and ability to influence employee perceptions favorably, thus increasing their well-being [55]. Identifying the mechanisms underlying the relationship between leadership and organizational commitment warrants further investigation. For instance, data suggest favorable communication between supervisors and subordinates concerning employment opportunities promotes work success [97].

In the present study, firm trust in supervisors is claimed to moderate psychological well-being and job satisfaction, which in turn leads to better job performance. First, we posit that supervisor social support, whereby employees feel free to discuss problems and ask for guidance, will serve as a stress buffer for lowering the negative impact of work stressors on psychological well-being. Next, supervisors’ leadership and attention should, by increasing well-being, yield improved job performance outcomes [92].

Thus, the following hypothesis is suggested:

**H3:**  *Trust in a supervisor moderates the relationship between psychological well-being and job satisfaction.*

The vast amount of research highlights the positive relationship between psychological well-being and high job performance [98,99]. Furthermore, Wright and Cropanzano (2000) posit that psychological well-being is associated with job performance, assuming that satisfied workers yield higher results when happiness is operationalized in terms of psychological well-being [100]. Psychological well-being is essential in forming personal responsibility behavior [101]. People with greater psychological well-being have higher intellectual performance, greater achievement, and better resilience when dealing with challenging circumstances [102,103]. Moreover, an employee with enhanced emotion regulation experiences increased satisfaction from carrying out responsibilities [104].

Previous research indicated that job satisfaction significantly mediates the relationship between psychological well-being and job performance [105]. Job satisfaction boosts employee performance through key psychosocial resources, such as intellectual capital. Well-being was previously associated with psychological capital and performance [106]. Satisfied employees are engaged, creative, and productive, and job satisfaction contributes to psychological well-being [107].

Job satisfaction and psychological well-being are often considered measurements of employees’ subjective happiness (e.g., affective appraisals of each attribute to their job, be it positive or negative). Due to its context dependency, job satisfaction is often operationalized in cognitive terms, and happiness pertaining to psychological well-being is a much broader and much more general concept, with primarily effective validation. Employees’ effectiveness and job performance are directly associated with the perception of satisfaction, content and autonomy, life balance, and happiness, which are the many constituents of psychological well-being. Employees who perceive their job as satisfactory and stimulating are likely to perform better than those who feel distressed, conflicted, unsatisfied, and unbalanced, or lacking psychological functionality [100].

Thus, the following hypothesis is suggested:

**H4:**  *The relationship between psychological well-being and job performance is mediated by job satisfaction.*

## 3. Materials and Methods

### 3.1. Participants and Procedure

A survey strategy was employed in the study. An online questionnaire was administered to employees of Bahraini enterprises. This study was conducted according to the guidelines of the Declaration of Helsinki and was approved by the Ethical Review Board of Zagreb’s School of Economics and Management under the approval code 1005. The research design was cross-sectional, taking place during a period when remote work had not yet become dominant, and the pandemic and post-pandemic context was still emerging. Consequently, the study captures perceptions and attitudes before remote work and post-pandemic contexts significantly influenced workplace dynamics.The convenience sampling technique was used to select participants. In agreement with the company managers, a link to an online questionnaire was sent to 400 white-collar workers. A total of 319 questionnaires were completed, demonstrating a high response rate and significant interest in the study. After eliminating incomplete answers, 277 were considered for analysis, ensuring the data’s quality and reliability.

Demographic information, including age, sex, work experience, industry, and education, was collected to provide a comprehensive understanding of the participant pool. A total of 64.9% of the analyzed respondents were female, and 36.1% were male, reflecting the gender distribution in the sample. Regarding age, 2 percent of participants were older than 55 years, 29.9 percent were aged 35 to 44 years, and 72.1 percent were aged between 24 and 35 years, indicating a relatively young workforce.

Almost half of the participants had less than five years of work experience, while 32.9 percent had 6 to 10 years of tenure, showcasing a mix of early and mid-career professionals. More than half held a bachelor’s degree, 20.2% had secondary education, and 1% had a Ph.D. degree, illustrating the educational diversity among the respondents.

Most of the surveyed individuals were employed in manufacturing (21.4%) or marketing and sales (20.3%), while a large portion of the sample population worked in accounting, HR, and administration. This distribution highlights the varied sectors represented in the study, providing a broad perspective on the white-collar workforce in Bahrain.

### 3.2. Measurements

The questionnaire consisted of Likert scales measuring psychological well-being, job performance, job satisfaction, and trust in the supervisor. Each scale was chosen for its reliability and validity in previous research. The questionnaire is in the Appendix A.

#### 3.2.1. Psychological Well-Being

The General Health Questionnaire-12 (GHQ-12) scale, developed by Goldberg and Williams (1991), was used to evaluate psychological well-being [108]. This scale consists of 12 items and measures various aspects of mental health, including anxiety, depression, social dysfunction, and loss of confidence. Respondents rated each item on a five-point scale ranging from “Strongly disagree” (1) to “Strongly agree” (5). The GHQ-12 is widely used in occupational health research and has demonstrated strong internal consistency.

#### 3.2.2. Job Satisfaction

Job satisfaction was measured using the Minnesota Satisfaction Questionnaire (MSQ) developed by Weiss et al. (1967) [109]. This scale assesses both intrinsic and extrinsic job satisfaction. Intrinsic satisfaction relates to the nature of the job itself, such as the tasks performed and the sense of achievement, while extrinsic satisfaction pertains to external factors such as pay, working conditions, and company policies. The MSQ includes 20 items rated on a five-point scale from “Very dissatisfied” (1) to “Very satisfied” (5). 

#### 3.2.3. Job Performance

A job performance scale developed by Williams and Anderson (1991) was adopted to measure employees’ in-role performance [110]. This scale consists of seven items that evaluate how well employees perform their job duties as specified in their job descriptions. Items such as “Adequately completes assigned duties” and “Fulfills responsibilities specified in job description” were included. Respondents rated their performance on a five-point scale ranging from “Strongly disagree” (1) to “Strongly agree” (5). The scale has been widely used in organizational research and is known for its robust psychometric properties.

#### 3.2.4. Trust in Supervisor

The Trust in Supervisor Scale, adopted from Robinson and Rousseau (1994), consists of seven items measuring the level of trust employees have in their supervisors [111]. This scale captures employees’ perceptions of their supervisors’ integrity, reliability, and fairness. Items include statements such as “I can trust my supervisor to keep my best interests in mind” and “My supervisor is always honest and truthful.” Each item was rated on a five-point scale from “Strongly disagree” (1) to “Strongly agree” (5). 

## 4. Results

### Path Analysis and Confirmatory Factor Analysis

We examined complex variables consisting of several items, tested the goodness of fit, and performed confirmatory factor analysis (CFA) using IBM SPSS AMOS version 23. Furthermore, structural equation modeling (SEM) was applied. CFA was performed to confirm the hypothesized research model and test the chosen factors. All the variables of psychological well-being, trust in the supervisor, job satisfaction, and job performance were placed in the four-factor model to be further examined in the measurement testing. CFA showed that the measurement model requires modifications by cleaning the items’ data due to insufficient factor loading. Both the visual measurement and standardized regression weight tables were used to identify the elements for removal.

All the indices except for the CFI verified good model fit (χ^2^/*df* = 2.699; CFI = 0.741; SRMR = 0.075; RMSEA = 0.078). A Bollen–Stine *p* < 0.05 indicated a poor fit. However, removing low-loading factors with an estimated value of less than 0.04 and above −0.04 improved the CFI to 0.769, still indicating poor marginal fit.

Modification of indices was performed by correlating high within-item errors in Job Satisfaction and Job Performance. Removal of items with residual covariance values greater than two resulted in significant improvements in the Bollen–Stine index (*p* > 0.05) and CFI, where the confirmatory factor index reached an acceptable level (CFI = 0.901). Other indicators illustrated the excellent fit of the model (χ^2^/*df* = 1.802; SRMR = 0.057; RMSEA = 0.054). A convergent validity check was performed through the average variance extracted (AVE) value. All the variables had good CR values above 0.7. The discriminant validity presented by the maximum shared variance (MSV) also showed acceptable validity. There are significant positive correlations between psychological well-being (PSW), job performance (JP), trust in supervisor (TS), and JS (Job Satisfaction), indicating that higher values in these variables are associated with higher job performance (Table 1).

The structural model, which included a direct relationship between psychological well-being, job performance, job satisfaction, and job satisfaction and job performance, showed an excellent fit (χ^2^/*df* = 1.945; CFI = 0.901; SRMR = 0.059; RMSEA = 0.059).

**Figure 2:**
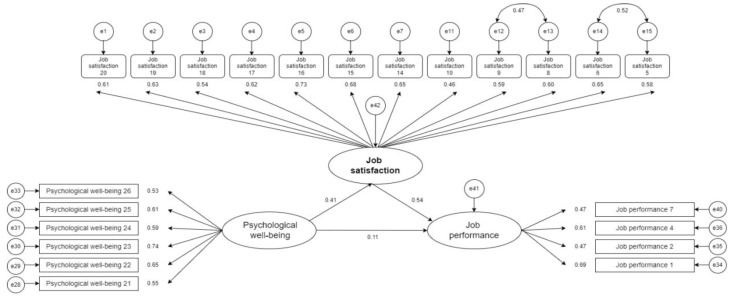
Structural equation modeling (SEM) of direct effects.

The hypothesized relationships were tested via structural equation modeling (Figure 2). For every increase in the raw score for psychological well-being, there was an increase of 0.571 for the raw score for job satisfaction (β = 0.41). Similarly, there is an increase of 0.495 in the raw score unit for job performance in relationship to job satisfaction (β = 0.54). Both relationships are highly significant. For psychological well-being predicting job performance, a direct connection between them was not confirmed; the raw coefficient is equal to 0.145 with β = 0.11, but the *p*-value has not reached the desired level of significance. All the statistical indicators are presented in Table 2.

Hypotheses H1 and H2, stating that psychological well-being has an impact on job satisfaction and that job satisfaction has a positive effect on job performance, were confirmed.

**Figure 3:**
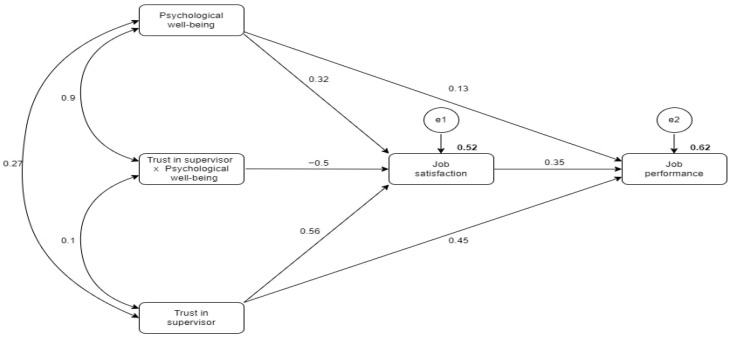
The moderating effect of trust in supervisor.

Hypothesis 3, stating that the impact of psychological well-being on job satisfaction increases with increasing trust in the supervisor, was checked via interaction effect testing. The unstandardized and standardized values are negative (β = −0.053), with a *p*-value of 0.207, indicating the insignificance of the relationship. The moderation effect of trust in a supervisor on the relationship between psychological well-being and job satisfaction has not been identified, thus rejecting hypothesis 3. Finally, the mediating impact of job satisfaction on the relationship between psychological well-being and job performance was tested by observing first the direct effects between variables and then the changes in the interaction if job satisfaction was included in the model. Hence, the direct effect of psychological well-being on job performance had a positive impact, with β = 0.113 and *p* > 0.05, indicating the insignificance of the direct relationship. However, the significance level rose considerably when job satisfaction was introduced into the relationship model (Figure 3). Hypothesis 4 states that the relationship between psychological well-being and job performance mediated by job satisfaction is confirmed, with β = 0.221 and <0.01.

## 5. Discussion

The current study examined the degree to which psychological well-being and trust are key for increasing perceived job satisfaction, thereby improving job performance. To achieve this objective, we have integrated Maslow’s Theory of Needs and Conservation of Resources Theory into a single framework, gaining insights into the complex interdependencies between psychological well-being, job satisfaction, trust, and performance. These approaches have been influential in the field because of their robustness and great predictive and explanatory power, as substantiated by our results.

A challenging problem that arises in organizational psychology studies is how to improve employee well-being and motivate personnel to sustain productivity in the wake of the global crisis threatening health and livelihoods [76,112,113]. In the post-pandemic era, many organizations have already undergone significant restructuring and digital transformation, which was not without its challenges. Compromised work–life balance, layoffs, role conflict, fear of employment, burnout, retraining, and remote work imposed a significant strain on the employees [76]. At the time this study was written, impairment due to prolonged exposure to psychosocial and economic stressors was exacerbated by a shift in technological means and the introduction of AI tools in organizational settings, with full implications expected to emerge over time [1,2]. Our research yielded results that corroborate previous positive relationships among well-being, satisfaction, trust, and performance. Our insights advocate the implementation of mindfulness and wellness initiatives to create a supportive organizational culture that may empower individuals and facilitate the transition.

A substantial organizational effort was invested in studying the motivations and drivers of employee productivity and how they relate to job satisfaction and individual and organizational well-being. Organizational management’s failure to create a stimulating and encouraging climate or mishandling of work conflicts can deprive a company of its most valuable asset—employee intelligence [114]. Thus, it is crucial to comprehend the underlying factors conducive to psychological well-being and satisfaction, as insights into determinants will aid organizations in the development of managerial policies and practices promoting health, self-actualization, and balance. It is essential to identify psychological complacency drivers, as satisfied and fulfilled workers yield better outcomes [91,115]. Scholars call for the immediate introduction of employee-friendly HR strategies to improve psychological well-being and enhance performance [116]. As evidenced by previous literature, post-pandemic changes in operational activities and remote policy have had a significant impact on psychological well-being, most noticeable in the quality of work–life [117] and work–life balance [118]. In the pandemic context, especially concerning the fear of COVID-19, job satisfaction and positive organizational outcomes were found to be related to leadership [119], supervisor–employee dynamics, such as rewards, uplifting skills, and communication [11].

In our study, psychological well-being was positively associated with job satisfaction. Workers exhibiting a greater degree of well-being are more flexible, resistant, and adaptive, and they possess a greater capacity to cope with challenges. The psychologically empowered and resilient workers display greater mental toughness [83]. Also, in the context of major uncertainty such as COVID-19, work–life integration was essential for psychological well-being, and this in turn boosts job satisfaction [105]. In line with the research of Doksaribu et al. (2022) and Dreer B. (2024), our results match the prior empirical evidence [120,121]. A similar conclusion was reached by Yang and Jo (2022) and Putra et al. (2024) [78,79]. Similarly, Ortan et al. (2021) found that facets of psychological well-being, such as self-efficacy and positive emotions, lead to job satisfaction [122].

The study results confirm the impact of job satisfaction on job performance. Our results are in line with those of previous studies [88,90]. We corroborated the results of Riyanto et al. (2021), who stated that motivation and job satisfaction have an effect on employee performance [6]. Job satisfaction is shaped by attitudes toward different organizational aspects, such as promotion, benefits, appreciation, organizational climate, nature of the job, and feelings toward other team members and overall communication [123].

Previous studies on the benefits and impacts of trust in supervisors on job satisfaction provided grounds for theorizing that trust in supervisors will intensify this association [92,93]. Contrary to our assumption, our results show no significant moderating effect. Some authors stress the dynamic nature of trust in the supervisor, arguing that it is less static and more variable-contingent on external environmental circumstances [124]. The fact that trust in supervisors was not significant in the Bahrain sample might be attributed to culture. Moreover, different leadership styles have disparate effects on employees’ well-being and job satisfaction [125]. For instance, Wang et al. (2022) stressed the adverse effects of abusive supervision on employee well-being [126]. If respondents are subjected to authoritative leadership, this would decrease employees’ autonomy and hinder self-developmental opportunities [22], thus impeding social well-being [127]. Our results indicate that psychological well-being significantly positively effects employee job satisfaction as presumed by SDG 3, and job satisfaction mediates the relationship between psychological well-being and job performance (SDG 8).

The mediation test results confirmed that job satisfaction mediates the relationship between psychological well-being and job performance. Satisfied workers who consider their job rewarding tend to be more psychologically balanced [107]. They are positive and tend to direct this energy and enthusiasm to the task at hand. Furthermore, the greater the psychological well-being and satisfaction are, the more focused and creative workers become [128]. Therefore, job satisfaction impacts job performance, and greater job performance, in turn, further stimulates satisfaction. Job satisfaction leads to greater physical and psychological health [129].

This research has significant implications for working conditions requiring greater autonomy and versatility to enhance organizational efficiency. The study emphasizes the crucial role of workplace conditions in improving staff contentment and happiness within the work setting, potentially contributing to more beneficial outcomes for the enterprise. Both psychological well-being and job satisfaction are found to contribute to job performance, and this is consistent with SDG 8—Decent work and economic growth. This project aims to develop an overarching framework to promote development-oriented policies that support productive activities [24,85].

State administrators and decision-makers should give closer consideration to creating employee-friendly working conditions to achieve greater operational performance. Additionally, the research offers valuable insight into organizational psychology and management research in the Middle East context.

### Limitations of the Study and Future Studies

The sample size was limited, and the use of convenience sampling methods reduced the generalizability of the research outcomes. Thus, it would be relevant to replicate the study in a different context, such as another country, or a specific business sector. Future research could evaluate the effect of individual and administrative influences on employee satisfaction and performance. As the focal point of this study was not implication of the pandemic on the work dynamics, future studies should investigate this area in more detail. At the onset of the pandemic, scholars and organizational scientists have found a detrimental effect of fear of COVID-19 on employee performance and job satisfaction [130,131,132]. Fear of COVID-19 has significantly impacted organizational strategy, accompanied by major digital transformation, thus imposing changes in managerial dynamics [133] and consequently job satisfaction [117]. Furthermore, the introduction of AI-based software and tools in the organizational environment requires a detailed commitment to devising effective adaptive practices [132]. For instance, there has been a major shift in structural changes, AI-enabled workplace culture, and employee satisfaction [134]. Accordingly, future studies could focus on the job satisfaction–job performance nuances in this new context.

## 6. Conclusions

As was previously hinted at by the catchphrase “happy worker–productive worker”, successful organizations stimulate outstanding achievements by yielding better performance. These employ all available resources, from counseling, team building, mentorship programs, support policies, and incentives, as these resources are crucial for overall organizational success. This paper considers what we assume to be the central stimulators of productive working behavior, namely, psychological well-being, job satisfaction, and job performance. Additionally, we introduce a construct of trust in supervisors into our analysis, which we expect to have a positive effect on increasing the strength of the relationship between psychological well-being and job satisfaction.

We have answered the call to provide empirical support for the beneficial work-related merits of psychological balance for employees’ productivity. We constructed a research model examining employees’ psychological well-being in terms of job-related satisfaction motives to establish a connection with job performance. We evaluated the psychological well-being-job performance process by introducing the concept of job satisfaction, i.e., employees’ favorable perceptions of autonomy, content, efficacy and fulfillment within an organization. Furthermore, we solidify our model by adding a widely discussed variable, trust in supervisor, to test the moderating effect. This research provides the groundwork for improving the study of job satisfaction and job performance in a Middle East business context.

## Figures and Tables

**Table 1:** Correlation matrix.

Variable	JP		PSW		TS		JS
1. JP	—						
	—						
	—						
2. PSW	277		—				
	0.416	***	—				
	<0.001		—				
3. TS	277		277		—		
	0.706	***	0.266	***	—		
	<0.001		<0.001		—		
4. JS	277		277		277		—
	0.700	***	0.474	***	0.643	***	—
	<0.001		<0.001		<0.001		—

*** *p* < 0.001.

**Table 2:** Summary of Structural Model Estimates, Parameter Estimates.

Predictor	Outcome	SRW	USRW	S.E.	C.R.	*p*-Value
PSW	JS	0.409	0.571	0.12	4.76	***
JS	JP	0.541	0.495	0.09	5.53	***
PSW	JP	0.113	0.145	0.107	1.347	0.178
JS	Job.Satisf16	0.729	1.121	0.117	9.565	***
JS	Job.Satisf15	0.679	1.081	0.119	9.09	***
JS	Job.Satisf14	0.653	1.034	0.117	8.832	***
JS	Job.Satisf10	0.455	0.717	0.109	6.594	***
JS	Job.Satisf9	0.587	0.954	0.117	8.13	***
JS	Job.Satisf8	0.595	0.964	0.117	8.221	***
JS	Job.Satisf6	0.649	1.073	0.122	8.784	***
JS	Job.Satisf5	0.564	0.839	0.107	7.858	***
PSW	Psycho.Well.B1	0.554	1	-	-	-
PSW	Psycho.Well.B2	0.646	1.181	0.157	7.51	***
PSW	Psycho.Well.B3	0.738	1.303	0.162	8.038	***
PSW	Psycho.Well.B4	0.594	1.035	0.145	7.137	***
PSW	Psycho.Well.B5	0.606	1.067	0.148	7.228	***
PSW	Psycho.Well.B6	0.534	0.965	0.145	6.652	***
JP	Job.Perform4	0.61	1.026	0.145	7.065	***
JP	Job.Perform2	0.466	0.778	0.132	5.908	***
JP	Job.Perform1	0.686	1	-	-	-
JP	Job.Perform7	0.471	0.743	0.125	5.958	***
JS	Job.Satisf17	0.616	0.982	0.116	8.451	***
ZPSW	ZJS	0.32	0.32	0.044	7.333	***
TS_x_PWB	ZJS	−0.053	−0.051	0.04	−1.263	0.207
ZJS	ZJP	0.352	0.352	0.053	6.577	***
ZTS	ZJP	0.445	0.445	0.049	9.118	***
ZPSW	ZJP	0.131	0.131	0.042	3.081	0.002

*** *p* < 0.001. PSW—psychological well-being, JS—job satisfaction, TS—trust in supervisor, JP—job performance.

## Data Availability

The raw data supporting the conclusions of this article will be made available by the authors on request.

## Appendix A

Psychological Well-being (GHQ-12)

Please rate the following statements concerning the past few weeks on a scale from (1) Strongly disagree to (5) Strongly agree.

1. I have been able to concentrate on whatever I’m doing.

2. I have lost much sleep over worry.

3. I have felt that I was playing a useful part in things.

4. I have felt capable of making decisions about things.

5. I have felt constantly under strain.

6. I have felt I couldn’t overcome my difficulties.

7. I have been able to enjoy my normal day-to-day activities.

8. I have been able to face up to problems.

9. I have been feeling unhappy or depressed.

10. I have been losing confidence in myself.

11. I have been thinking of myself as a worthless person.

12. I have been feeling reasonably happy, all things considered.

Job Satisfaction

Rate your satisfaction from (1) Very dissatisfied to (5) Very satisfied.

1. Being able to keep busy all the time.

2. The chance to work alone on the job.

3. The chance to do different things from time to time.

4. The chance to be ‘somebody’ in the community.

5. The way my boss handles employees.

6. The competence of my superior in making decisions.

7. Being able to do things that do not go against my conscience.

8. The way my job provides for steady employment.

9. The chance to do things for other people.

10. The chance to tell people what to do.

11. The chance to do something that makes use of my abilities.

12. The way company policies are put into practice.

13. My pay and the amount of work I do.

14. The chance for advancement on this job.

15. The freedom to use my own judgment.

16. The chance to try my own methods of doing my job.

17. The working conditions.

18. The way my co-workers get along with each other.

19. The praise I get for doing a good job.

20. The feeling of accomplishment I get from the job.

Trust in Supervisor

Rate the following statements about your supervisor from (1) Strongly disagree to (5) Strongly agree.

1. I can trust my supervisor to keep my best interests in mind.

2. My supervisor is always honest and truthful.

3. My supervisor treats me fairly and justly.

4. I feel confident that my supervisor will always keep promises made to me.

5. I can rely on my supervisor not to take advantage of me.

6. My supervisor is trustworthy.

7. My supervisor is dependable.

Job Performance

Rate your agreement with the following statements about your job performance from (1) Strongly disagree to (5) Strongly agree.

1. Adequately completes assigned duties.

2. Engages in activities that will directly affect his or her performance evaluation.

3. Fails to perform essential duties. (R)

4. Fulfills responsibilities specified in job description.

5. Meets formal performance requirements of the job.

6. Neglects aspects of the job he or she is obliged to perform. (R)

7. Performs tasks that are expected of him or her.

## References

[1] Trenerry B., Chng S., Wang Y., Suhaila Z.S., Lim S.S., Lu H.Y., Oh P.H. (2021). Preparing workplaces for digital transformation: An integrative review and framework of multi-level factors. Front. Psychol..

[2] Dąbrowska J., Almpanopoulou A., Brem A., Chesbrough H., Cucino V., Di Minin A., Giones F., Hakala H., Marullo C., Mention A.-L. (2022). Digital transformation, for better or worse: A critical multi-level research agenda. RD Manag..

[3] DiMaria C.H., Peroni C., Sarracino F. (2020). Happiness matters: Productivity gains from subjective well-being. J. Happiness Stud..

[4] Karapinar P.B., Camgoz S.M., Ekmekci O.T. (2019). Employee well-being, workaholism, work–family conflict and instrumental spousal support: A moderated mediation model. J. Happiness Stud..

[5] Bocean C.G., Popescu L., Varzaru A.A., Avram C.D., Iancu A. (2023). Work-Life Balance and Employee Satisfaction during COVID-19 Pandemic. Sustainability.

[6] Riyanto S., Endri E., Herlisha N. (2021). Effect of work motivation and job satisfaction on employee performance: Mediating role of employee engagement. Probl. Perspect. Manag..

[7] Susanto P.C., Sawitri N.N., Suroso S. (2023). Determinant Employee Performance and Job Satisfaction: Analysis Motivation, Path Career and Employee Engagement in Transportation and Logistics Industry. Int. J. Bus. Appl. Econ..

[8] Rahmawanti N.P. (2014). Pengaruh Lingkungan Kerja Terhadap Kinerja Karyawan (Studi Pada Karyawan Kantor Pelayanan Pajak Pratama Malang Utara). Doctoral Dissertation.

[9] Hartika A., Fitridiani M., Asbari M. (2023). The effect of job satisfaction and job loyalty on employee performance: A narrative literature review. J. Inf. Syst. Manag. (JISMA).

[10] Chowhan J., Pike K. (2023). Workload, work–life interface, stress, job satisfaction and job performance: A job demand–resource model study during COVID-19. Int. J. Manpow..

[11] Nemțeanu M.S., Dinu V., Pop R.A., Dabija D.C. (2022). Predicting job satisfaction and work engagement behavior in the COVID-19 pandemic: A conservation of resources theory approach. E+M Èkon. Manag..

[12] Kundi Y.M., Aboramadan M., Elhamalawi E.M., Shahid S. (2020). Employee psychological well-being and job performance: Exploring mediating and moderating mechanisms. Int. J. Organ. Anal..

[13] Keefer P., Vlaicu R. (2024). Employee trust and performance constraints in public sector organizations. Eur. J. Political Econ..

[14] Zak P.J. (2017). The Neuroscience of Trust: Management behaviors that foster employee engagement. Harvard Business Review..

[15] Tavassoli T., Sunyer Torrents A. (2020). Employee work-life balance, satisfaction and burnout in Iran and Spain. Humanit. Soc. Sci. Rev..

[16] Edmondson A.C., Kramer R.M., Cook K.S. (2004). Psychological safety, trust, and learning in organizations: A group-level lens. Trust. Distrust Organ. Dilemmas Approaches.

[17] Bakker A.B., Hetland J., Olsen O.K., Espevik R. (2019). Daily strengths use and employee well-being: The moderating role of personality. J. Occup. Organ. Psychol..

[18] Tisu L., Lupșa D., Vîrgă D., Rusu A. (2020). Personality characteristics, job performance and mental health the mediating role of work engagement. Personal. Individ. Differ..

[19] Jia K., Zhu T., Zhang W., Rasool S.F., Asghar A., Chin T. (2022). The Linkage between Ethical Leadership, Well-Being, Work Engagement, and Innovative Work Behavior: The Empirical Evidence from the Higher Education Sector of China. Int. J. Environ. Res. Public Health.

[20] Sundberg J. (2023). Employee Autonomy Can Drive Company Profitability in Sourcing.

[21] Dudasova L., Vaculik M., Prochazka J., Svitavska P., Patton G. (2023). Causality of the satisfaction–performance relationship: A task experiment. Eur. J. Psychol..

[22] Turban D.B., Yan W. (2016). Relationship of eudaimonia and hedonia with work outcomes. J. Manag. Psychol..

[23] Salgado J.F., Blanco S., Moscoso S. (2019). Subjective well-being and job performance: Testing of a suppressor effect. Rev. Psicol. Trab. Organ..

[24] United Nations (2021). Sustainable Development Goals. https://unstats.un.org/sdgs/report/2021/.

[25] Madero-Gómez S.M., Rubio Leal Y.L., Olivas-Luján M., Yusliza M.Y. (2023). Companies could benefit when they focus on employee wellbeing and the environment: A systematic review of Sustainable Human Resource Management. Sustainability.

[26] Smith A., Gonzalez Smith D.T., Ogunwale A., Bhugra D., Buadze A., Ventriglio A., Liebrenz M. (2024). Geopsychiatry, the United Nations’ Sustainable Development Goals, and geopolitical challenges for global mental health. Int. J. Soc. Psychiatry.

[27] Mariappanadar S. (2024). Improving Quality of Work for Positive Health: Interaction of Sustainable Development Goal (SDG) 8 and SDG 3 from the Sustainable HRM Perspective. Sustainability.

[28] World Health Organization (2018). Mental Health at Work. https://www.who.int/news-room/fact-sheets/detail/mental-health-at-work.

[29] Mishra H., Venkatesan M. (2023). Psychological well-being of employees, its precedents and outcomes: A literature review and proposed framework. Manag. Labour Stud..

[30] Danilewicz D. (2020). The Concept of Well-being in Organisational Reality. Eduk. Ekon. I Menedżerów.

[31] Brown S.D., Dahill D., Karakilic E., King D., Misha P., Pirrioni S., Shipton H., Vedi P. (2020). Psychological Wellbeing and Safety in a Global Context: A Rapid Evidence Assessment.

[32] Hitesh M., Ahmed L.Z. (2021). Psychological Wellbeing of Employees. Int. J. Emerg. Technol. Innov. Res..

[33] Savchenko O.V., Lavrynenko D.G., Kononenko T.V. (2022). Psychological flexibility as a factor in staff’s psychological well-being. Organ. Psychol. Econ. Psychol..

[34] Gaddam R.P., Perwez S.K., Lathabhavan R., Padhy P. (2023). Well-Being of Organizational Leaders in the New Normal. Community Mental Health and Well-Being in the New Normal.

[35] Sajjad A., Shahbaz W. (2020). Mindfulness and Social Sustainability: An Integrative Review. Soc. Indic. Res..

[36] Dhanabhakyam M., Sarath M. (2023). Psychological wellbeing: A systematic literature review. Int. J. Adv. Res. Sci. Commun. Technol..

[37] Lévy-Garboua L., Montmarquette C. (2011). Demand. A Handbook of Cultural Economics.

[38] Fisher E. (2010). Media and New Capitalism in the Digital Age: The Spirit of Networks.

[39] Wilkes L., Doull M., Ng Chok H., Mashingaidze G. (2017). Developing a tool to measure the factors influencing nurses’ enjoyment of nursing. J. Clin. Nurs..

[40] Goetzel R.Z., Roemer E.C., Holingue C., Fallin M.D., McCleary K., Eaton W., Agnew J., Azocar F., Ballard D., Bartlett J. (2018). Mental health in the workplace: A call to action proceedings from the Mental Health in the Workplace—Public Health Summit. J. Occup. Environ. Med..

[41] Abawa A., Obse H. (2024). Organizational culture and organizational performance: Does job satisfaction mediate the relationship?. Cogent Bus. Manag..

[42] Paul D. (2024). Effects of Monetary and Non-Monetary Incentives on Employee’s Performance. Manag. J. Adv. Res..

[43] Bolatito A.O.S., Mohamoud Y.A. (2024). Reward Management and Employee Performance: A Review of Job Satisfaction in Somalia. TWIST.

[44] Ribeiro N., Gomes D., Oliveira A.R., Dias Semedo A.S. (2023). The impact of the work-family conflict on employee engagement, performance, and turnover intention. Int. J. Organ. Anal..

[45] Haji W.H., Madiistriyatno H., Widayati C.C., Usman M. (2021). The Influence Of Knowledge Management, Skill, And Attitude On Employee Performance. Dinasti Int. J. Digit. Bus. Manag..

[46] Rathi S.R., Islam A. (2024). Work-Life Balance and Job Satisfaction as Predictors of Job Performance among Bankers: A Cross-Sectional Study. Int. J. Indian Psychȯl..

[47] Kloutsiniotis P.V., Mihail D.M. (2020). The effects of high performance work systems in employees’ service-oriented OCB. Int. J. Hosp. Manag..

[48] Peng X., Lee S., Lu Z. (2020). Employees’ perceived job performance, organizational identification, and pro-environmental behaviors in the hotel industry. Int. J. Hosp. Manag..

[49] Tucker M.K., Jimmieson N.L., Bordia P. (2018). Supervisor support as a double-edged sword: Supervisor emotion management accounts for the buffering and reverse-buffering effects of supervisor support. Int. J. Stress Manag..

[50] Zeb A., Goh G.G.G., Javaid M., Khan M.N., Khan A.U., Gul S. (2023). The interplay between supervisor support and job performance: Implications of social exchange and social learning theories. J. Appl. Res. High. Educ..

[51] Sekhar C., Patwardhan M. (2023). Flexible working arrangement and job performance: The mediating role of supervisor support. Int. J. Product. Perform. Manag..

[52] Sen H.T., Yıldırım A. (2023). The relationship between nurses’ perceived organizational, supervisor and co-worker support, psychological well-being and job performance. J. Pak. Med. Assoc..

[53] Rofcanin Y., Wang S., Heras M.L., Taser D., Bosch M.J., Fındıklı M.A., Vallina A.S. (2023). Perceptions of support trickle down: Effects on energetic resources via psychological empowerment. Eur. Manag. Rev..

[54] Herr R.M., Li J., Angerer P. (2019). The synergistic effects of organizational justice and trust to supervisor on vagal tone: Preliminary findings of an empirical investigation. Int. J. Environ. Res. Public Health.

[55] Basit A.A. (2021). Trust in supervisor and job engagement: Mediating effects of psychological safety and felt obligation. Leadership and Supervision.

[56] Rousseau D.M., Sitkin S.B., Burt R.S., Camerer C. (1998). Not so different after all: A cross-discipline view of trust. Acad. Manag. Rev..

[57] Men L.R., Qin Y.S., Jin J. (2022). Fostering employee trust via effective supervisory communication during the COVID-19 pandemic: Through the lens of motivating language theory. Int. J. Bus. Commun..

[58] Lapointe É., Vandenberghe C. (2018). Trust in the supervisor and the development of employees’ social capital during organizational entry: A conservation of resources approach. Int. J. Hum. Resour. Manag..

[59] Skiba T., Wildman J.L. (2019). Uncertainty reducer, exchange deepener, or self-determination enhancer? Feeling trust versus feeling trusted in supervisor-subordinate relationships. J. Bus. Psychol..

[60] Yu T.W. (2022). The effects of organizational justice, trust and supervisor–subordinate guanxi on organizational citizenship behavior: A social-exchange perspective. Manag. Res. Rev..

[61] Damodaran A., Shulruf B., Jones P. (2017). Trust’versus ‘competency’in the workplace. Med. Educ..

[62] Silitonga K.A.A., Ahmad F., Simanjuntak C.W., Atrizka D. (2020). Exploring the nexus between the HR practices and work engagement: The mediating role of Job Demand. Syst. Rev. Pharm..

[63] Maslow A.H., Lowry R. (1973). A theory of human motivation. Dominance, Self-Esteem, Self-Actualization: Germinal Papers of A.H. Maslow.

[64] Hobfoll S.E. (1989). Conservation of resources: A new attempt at conceptualizing stress. Am. Psychol..

[65] Harter J.K., Schmidt F.L., Hayes T.L. (2002). Business-unit-level relationship between employee satisfaction, employee engagement, and business outcomes: A meta-analysis. J. Appl. Psychol..

[66] Hameli K., Çollaku L., Ukaj L. (2024). The impact of job burnout on job satisfaction and intention to change occupation among accountants: The mediating role of psychological well-being. Ind. Commer. Train..

[67] Mamaqi E. (2023). The Role of Trainings in the Development and Enhancement of Work Performance in the Public and Private Sector. Interdiscip. J. Res. Dev..

[68] Eljali A., Ameen A. (2020). Impact of the Training (Analysis, Design, and Development) on Employee Performance (Execution Skill, Knowledge, and Adapt to Work). Int. J. Sci. Res. Publ..

[69] Jung H.S., Hwang Y.H., Yoon H.H. (2023). Impact of Hotel Employees’ Psychological Well-Being on Job Satisfaction and Pro-Social Service Behavior: Moderating Effect of Work–Life Balance. Sustainability.

[70] Bessing B., Claflin S.B., Taylor B.V., Blizzard L., Honan C.A., van Dijk P., Kirk-Brown A., van der Mei I. (2022). Estimating the impact of work difficulties, work self-efficacy and work psychological safety on MS-related work productivity loss. Mult. Scler. J..

[71] Gulyamova S.T., Abdul Aziz S.F., Omar N.H., Mohd R.H. (2023). Workplace-Related Socioeconomic Issues Associated with Job Performance and Productivity among Employees with Various Impairments: A Systematic Literature Review. Soc. Sci..

[72] Riaz Z., Stankeviciute Ž., Pinzaru F. (2024). New Work Demands and Managing Employee Well-being in the Post-pandemic World. Front. Psychol..

[73] Radu C., Deaconu A., Kis I.A., Jansen A., Mișu S.I. (2023). New Ways to Perform: Employees’ Perspective on Remote Work and Psychological Security in the Post-Pandemic Era. Sustainability.

[74] Meyer B., Zill A., Dilba D., Gerlach R., Schumann S. (2021). Employee psychological well-being during the COVID-19 pandemic in Germany: A longitudinal study of demands, resources, and exhaustion. Int. J. Psychol..

[75] Harju L.K., Rokka J., Lopes M.M., Airoldi M., Raïes K. (2021). Employee well-being profiles during COVID-19 lockdown: A latent profile analysis of French and UK employees. Front. Psychol..

[76] Peters S.E., Dennerlein J.T., Wagner G.R., Sorensen G. (2022). Work and worker health in the post-pandemic world: A public health perspective. Lancet Public Health.

[77] Kim C.Y. (2021). Psychological well-being, knowledge management behavior and performance: The moderating role of leader-member exchange. Front. Psychol..

[78] Yang X., Jo W.M. (2022). Roles of work-life balance and trait mindfulness between recovery experiences and employee subjective well-being: A moderated mediation model. J. Hosp. Tour. Manag..

[79] Putra A.S.B., Kusumawati E.D., Kartikasari D. (2024). Psychological empowerment and psychological well-being as job performance mediators. J. Bus. Manag. Econ. Dev..

[80] Kurtuluş E., Kurtuluş H.Y., Birel S., Batmaz H. (2023). The effect of social support on work-life balance: The role of psychological well-being. Int. J. Contemp. Educ. Res..

[81] Susanto P.C., Supardi S., Parmenas N.H., Tannady H., Soehaditama J.P. (2023). Mini Review: Work-Life Balance, Psychological Structure, Employee Resilience, and Organization Commitment to Employee Wellbeing: Human Resource Management. Int. J. Psychol. Health Sci..

[82] Bayighomog S., Arasli H. (2022). Reviving employees’ essence of hospitality through spiritual wellbeing, spiritual leadership, and emotional intelligence. Tour. Manag..

[83] Lee M., Kim B. (2023). Effect of the employees’ mental toughness on organizational commitment and job satisfaction: Mediating psychological well-being. Adm. Sci..

[84] Upadhyaya C. (2014). Application of the Maslow’s hierarchy of need theory; impacts and implications on organizational culture, human resource and employee’s performance. Int. J. Educ. Manag. Stud..

[85] Sachs J., Kroll C., Lafortune G., Fuller G., Woelm F. (2021). The Decade of Action for the Sustainable Development Goals: Sustainable Development Report 2021.

[86] Diener E., Suh E.M., Lucas R.E., Smith H.L. (1999). Subjective well-being: Three decades of progress. Psychol. Bull..

[87] Yusnita N., Melyiatama M., Irawan T.T. (2023). The Effect of Work Environment on Performance through Job Satisfaction. Manag. J. Binaniaga.

[88] Khoso I., Raza A. (2023). Analysis of Individual Performance through Job Satisfaction: A Study of Faculty Members in Public Sector Universities in Sindh, Pakistan. Voyag. J. Educ. Stud..

[89] Valaei N., Rezaei S. (2016). Job satisfaction and organisational commitment. Manag. Res. Rev..

[90] Sanjaya M., Indrawati L. (2023). The Influence of Job Satisfaction, Work Motivation, and Employee Commitment on Employee Performance. Res. Manag. Account. (RIMA).

[91] Kumari P.B., Hemalatha A.A. (2018). Study on Impact of Employee Empowerment on Job Satisfaction with Reference to Ford Motor Private Ltd., Perungudi. Eurasian J. Anal. Chem..

[92] Herawati H., Setyadi D., Michael M., Hidayati T. (2023). The effect of workload, supervisor, and coworker supports on job performance through job satisfaction. Int. J. Financ. Econ. Bus..

[93] Kurniawan R., Anindita R. (2021). Impact of Perceived Supervisor Support and Rewards and Recognition toward Performance through Work Satisfaction and Employee Engagement in Employee Marketing Banks. Bus. Entrep. Rev..

[94] Macey W.H., Schneider B. (2008). The meaning of employee engagement. Ind. Organ. Psychol..

[95] Baqir M., Hussain S., Waseem R., Islam K.A. (2020). Impact of reward and recognition, supervisor support on employee engagement. Am. Int. J. Bus. Manag. Stud..

[96] Saks A.M. (2006). Antecedents and consequences of employee engagement. J. Manag. Psychol..

[97] Breevaart K., Bakker A., Hetland J., Demerouti E., Olsen O.K., Espevik R. (2014). Daily transactional and transformational leadership and daily employee engagement. J. Occup. Organ. Psychol..

[98] Nika F.A., Bashir I. (2023). Impact of Psychological Well-being on Employee Performance and Productivity. Productivity.

[99] Obrenovic B., Jianguo D., Khudaykulov A., Khan M.A.S. (2020). Work-family conflict impact on psychological safety and psychological well-being: A job performance model. Front. Psychol..

[100] Cropanzano R., Aguinis H., Schminke M., Denham D.L. (1999). Disputant reactions to managerial conflict resolution tactics: A comparison among Argentina, the Dominican Republic, Mexico, and the United States. Group Organ. Manag..

[101] Christopher J.C. (1999). Situating psychological well-being: Exploring the cultural roots of its theory and research. J. Couns. Dev..

[102] Min W., Jun G., Feng L. (2022). How Psychological Wellbeing and Digital Mental Health Services Intervene the Role of Self-Efficacy and Affective Commitment of University Students in Their Performance?. Front. Psychiatry.

[103] Trucchia S.M., Lucchese M.M., Enders J.E., Fernández R. (2013). Relationship between academic performance, psychological well-being, and coping strategies in medical students. Rev. Fac. Cienc. Médicas.

[104] Nieto M.A., Huang R.Y., Jackson R.A., Thiery J.P. (2016). EMT: 2016. Cell.

[105] Pudjiati P., ZA S.Z., AS D.L. (2023). Job Satisfaction and Psychological Well-Being as Employee Performance Factors in Educational Institutions. IJEBD Int. J. Entrep. Bus. Dev..

[106] Imran M., Shahnawaz M.G. (2020). PsyCap and performance: Wellbeing at work as a mediator. Asia-Pac. J. Manag. Res. Innov..

[107] Liona R.C., Yurniardi M.S. (2020). The contribution of work engagement and job satisfaction to workers’ psychological well-being. Humanitas.

[108] Goldberg D., Williams P. (1991). A Users Guide to the General Healthcare Questionnaire: GHQ. 1988.

[109] Weiss D.J., Dawis R.V., England G.W. (1967). Manual for the Minnesota satisfaction questionnaire. Minn. Stud. Vocat. Rehabil..

[110] Williams L.J., Anderson S.E. (1991). Job satisfaction and organisational commitment as predictors of organisational citizenship and in-role behaviors. J. Manag..

[111] Robinson S.L., Rousseau D.M. (1994). Violating the psychological contract: Not the exception but the norm. J. Organ. Behav..

[112] Chafi M.B., Hultberg A., Yams N.B. (2021). Post-pandemic office work: Perceived challenges and opportunities for a sustainable work environment. Sustainability.

[113] Bulińska-Stangrecka H., Bagieńska A. (2021). The role of employee relations in shaping job satisfaction as an element promoting positive mental health at work in the era of COVID-19. Int. J. Environ. Res. Public Health.

[114] Pathan M.S.K. (2022). The influence of organizational culture on employee commitment and turnover intentions. Int. Res. J. Manag. Soc. Sci..

[115] Pasulu M., Irfan A., Pahmi A.A., Thalib L. (2023). The Effect of Job Satisfaction and Work Motivation on Employee Performance through Work Discipline at the Regional Secretariat of East Luwu Regency. Acc. Financial Manag. J..

[116] Senbursa N. (2022). Employee-Friendly Human Resources Management Strategies in the New Age “Covid” Era. Navigating the New Normal of Business with Enhanced Human Resource Management Strategies.

[117] Rai G.D., Verma S. (2023). Quality of work life, fear of COVID-19, job satisfaction, and commitment: A moderated mediation model. Int. J. Product. Perform. Manag..

[118] Alfano V., Mariotti I., Marra M., Vecchione G. (2023). I want to break free: The influence of the COVID-19 pandemic on work–life balance satisfaction. Reg. Stud. Reg. Sci..

[119] Abolnasser M.S.A., Abdou A.H., Hassan T.H., Salem A.E. (2023). Transformational leadership, employee engagement, job satisfaction, and psychological well-being among hotel employees after the height of the COVID-19 pandemic: A serial mediation model. Int. J. Environ. Res. Public Health.

[120] Doloksaribu M.F., Lubis M.R., Ideyani N. (2022). Pengaruh Kesejahteraan Psikologis dan Iklim Organisasi terhadap Kepuasan Kerja. J. Educ. Hum. Hum. Soc. Sci. (JEHSS).

[121] Dreer B. (2024). Teachers’ well-being and job satisfaction: The important role of positive emotions in the workplace. Educ. Stud..

[122] Ortan F., Simut C., Simut R. (2021). Self-efficacy, job satisfaction and teacher well-being in the K-12 educational system. Int. J. Environ. Res. Public Health.

[123] Judge T.A., Zhang S.C., Glerum D.R. (2020). Job satisfaction. Essentials of Job Attitudes and Other Workplace Psychological Constructs.

[124] Bijlsma-Frankema K., Costa A.C. (2005). Understanding the trust-control nexus. Int. Sociol..

[125] Walsh M.M., Arnold K.A. (2020). The bright and dark sides of employee mindfulness: Leadership style and employee well-being. Stress Health.

[126] Wang I.-A., Lin S.-Y., Chen Y.-S., Wu S.-T. (2022). The influences of abusive supervision on job satisfaction and mental health: The path through emotional labor. Pers. Rev..

[127] Zhou X., Rasool S.F., Yang J., Asghar M.Z. (2021). Exploring the relationship between despotic leadership and job satisfaction: The role of self-efficacy and leader–member exchange. Int. J. Environ. Res. Public Health.

[128] Aldabbas H., Pinnington A., Lahrech A. (2023). The influence of perceived organizational support on employee creativity: The mediating role of work engagement. Curr. Psychol..

[129] Faragher R., Harle R. (2015). Location fingerprinting with bluetooth low energy beacons. IEEE J. Sel. Areas Commun..

[130] Nagarajan R., Swamy R.A., Reio T.G., Elangovan R., Parayitam S. (2022). The COVID-19 impact on employee performance and satisfaction: A moderated moderated-mediation conditional model of job crafting and employee engagement. Hum. Resour. Dev. Int..

[131] Çağış Z.G., Yıldırım M. (2023). Understanding the effect of fear of COVID-19 on COVID-19 burnout and job satisfaction: A mediation model of psychological capital. Psychol. Health Med..

[132] Han Z., Wang D., Jiang C., Zhang Y. (2023). Enhancing Employee Job Satisfaction Responding to COVID-19: The Role of Organizational Adaptive Practices and Psychological Resilience. Psychol. Res. Behav. Manag..

[133] Kadriu F., Kleemann J., Sorg N., Härting R., Reichstein C. (2023). Impact of Telework on Employee Satisfaction During the COVID-19 Crisis. KES International Symposium on Agent and Multi-Agent Systems: Technologies and Applications.

[134] Singh R., Tarkar P. (2022). Future of work: How Artificial Intelligence will change the dynamics of work culture and influence employees work satisfaction post-COVID-19. Proceedings of International Conference on Communication and Artificial Intelligence: ICCAI 2021.

